# The association between the nicotinic acetylcholine receptor α4 subunit gene (*CHRNA4*) rs1044396 and Internet gaming disorder in Korean male adults

**DOI:** 10.1371/journal.pone.0188358

**Published:** 2017-12-14

**Authors:** Jo-Eun Jeong, Je-Keun Rhee, Tae-Min Kim, Su-Min Kwak, Sol-hee Bang, Hyun Cho, Young-Hoon Cheon, Jung Ah Min, Gil Sang Yoo, Kyudong Kim, Jung-Seok Choi, Sam-Wook Choi, Dai-Jin Kim

**Affiliations:** 1 Department of Psychiatry, Seoul St. Mary’s Hospital, College of Medicine, The Catholic University of Korea, Seoul, Republic of Korea; 2 Catholic Cancer Research Institute, College of Medicine, The Catholic University of Korea, Seoul, Republic of Korea; 3 Department of Medical Informatics, College of Medicine, The Catholic University of Korea, Seoul, Republic of Korea; 4 Department of Psychiatry, Incheon Chamsarang Hospital, Incheon, Republic of Korea; 5 Department of Psychiatry, Incheon St. Mary’s Hospital, College of Medicine, The Catholic University of Korea, Seoul, Republic of Korea; 6 Department of Psychiatry, Uijeongbu St. Mary’s Hospital, College of Medicine, The Catholic University of Korea, Seoul, Republic of Korea; 7 Department of Psychiatry, SMU-SNU Boramae Medical Center, Seoul, Republic of Korea; 8 Department of Psychiatry, Seoul National University College of Medicine, Seoul, Republic of Korea; 9 Department of Psychiatry, True Mind Mental Health Clinic, Seoul, Republic of Korea; Ariel University, ISRAEL

## Abstract

The primary aim of this study was to investigate the genetic predisposition of Internet gaming disorder (IGD), and the secondary aim was to compare the results to those of alcohol dependence (AD). Two independent case-control studies were conducted. A total of 30 male participants with IGD, diagnosed according to the 5^th^ edition of the *Diagnostic and Statistical Manual of Mental Disorders* (DSM-5) criteria, and 30 sex-matched controls participated in study 1. We designed targeted exome sequencing (TES) to test for 72 candidate genes that have been implicated in the pathogenesis of addiction. The genes included seven neurotransmitter (dopamine, serotonin, glutamate, r-aminobutyric acid (GABA), norepinephrine, acetylcholine, and opioid) system genes. A total of 31 male in-patients with AD and 29 normal male controls (NC) were enrolled in study 2. The same 72 genes included in study 1 and ten additional genes related to alcohol-metabolic enzyme were selected as the target genes, and we identified the genetic variants using the same method (TES). The IGD group had a lower frequency of the T allele of rs1044396 in the nicotinic acetylcholine receptor alpha 4 subunit (*CHRNA4*), and this variant represents a protective allele against IGD. However, we did not find a significant difference in the polymorphisms of the 72 genes that encode neurotransmitter systems between the AD and NC groups. This study demonstrated that rs1044396 of *CHRNA4* was significantly associated with IGD.

## Introduction

As the Internet becomes an essential part of everyday life, the number of Internet users is increasing worldwide [1]. In reports about Internet usage in 2016, approximately 89.9% of the population of South Korea [2] and 89.0% of North America [3] are using the Internet. As Internet use grows, concern about problematic Internet use is also increasing. Although there is no consensus as to what constitutes ‘problematic Internet use (PIU)’, it has been proposed that PIU refers to excessive Internet use that interferes with daily life [4]. Additionally, some studies have suggested that the Internet itself is not the issue, but rather the type of medium and diversity of contents, such as gambling, pornography, and gaming, that are concerning [5–7]. A previous study has also shown that there were differences between individuals with PIU who only played video games and those who only watched pornography [8]. That means that PIU should be replaced by problems more specifically pertaining to these behaviors. In this context, the categorization of PIU into more specific forms, such as Internet gaming disorder (IGD), has been proposed.

IGD is newly listed in Section III, Conditions for Further Study, of the 5^th^ edition of the *Diagnostic and Statistical Manual of Mental Disorders* (DSM-5) [9] and is currently proposed as the formal diagnostic criteria for gaming disorder in the International Classification of Diseases (ICD) -11 [10]. The DSM-5 proposed that IGD is manifested by persistent or recurrent gaming behavior characterized by impaired control over gaming, leading to clinically significant impairment or distress.

Furthermore, growing evidence suggests that IGD resembles substance addiction in many domains, including phenomenology, co-morbidity, and neurobiological mechanisms [11]. In terms of genetic features, IGD associations with personality traits, such as sensation-seeking [12], impulsivity [13], and self-control [14], have been identified. In addition, several association studies showed that the Taq1A1 allele of the dopamine receptor gene (*DRD2*) [15], the methionine variant of dopamine degradation enzyme gene (*COMT*) [15], short allelic variants of the serotonin transporter gene (*5-HTTLPR*) [16], and polymorphisms of the nicotinic acetylcholine receptor gene (*CHRNA4*) [17] and neurotrophic tyrosine kinase type 3 receptor gene (*NTRK3*) [18] were associated with PIU. These personality traits and polymorphisms have also been shown to influence diverse substance addictions [19–24]. Although no genome wide association studies (GWAS) have been reported for IGD until now, two twin studies have shown that genetic influence on PIU was estimated at between 48% and 66% [11, 25]. These figures are comparable to the heritability of substance addictions [26, 27].

These similarities suggest that it is likely that IGD and substance addictions share a common biological underpinning, which may also be partially influenced by similar genetic factors. Previous studies indicate that multiple neurotransmitter systems may play a role in the development of substance and non-substance addictions [28, 29]. Specifically, the mesolimbic dopamine (DA) pathway is implicated in the development of addictions, and this system interacts with numerous other neurotransmitters including serotonergic, glutamatergic, r-aminobutyric acid (GABA), adrenergic, cholinergic, and opioid pathways [30, 31].

We performed a case-control association study of addiction-associated neurotransmitter system genes in both IGD and AD, which represented substance addiction. Additionally, rather than using the existing GWAS, we used targeted exome sequencing (TES), a new approach for identifying genetic variants. GWAS can cover the entire genome, but it requires a very large sample size to obtain the necessary statistical power. Using the exomes, the protein-coding regions, is more efficient since it constitutes only ~1% of the human genome, but harbors much of the functional variation [32]. TES enables focusing on specific candidate genes and achieves a very high depth of coverage (100X coverage or greater) of the regions of interest [33].

The primary aim of the study was to investigate the genetic predisposition of IGD. The secondary aim was to compare the results to those in AD. Study 1 was a case-control study of IGD and Study 2 was an independent case-control study of AD. Both studies included the same basic set of candidate genes. Study 2 added the alcohol-metabolizing enzyme genes, which were consistently reported in the results of a previous GWAS of an East Asian sample [34, 35].

## Materials and methods

### Study 1 (Case-control study of IGD)

#### Participants

The IGD patients were recruited from the addiction clinics of Seoul St. Mary’s Hospital and Boramae Medical Center and were diagnosed according to the DSM-5 criteria for IGD by clinically experienced psychiatrists (D.J.K. and J.S.C.). For homogeneity, the participants were restricted to male adults over 18 years of age. Based on previous studies, men are more likely than women to play [36] and be addicted to Internet games [37]. In addition, there are gender differences in the characteristics of gaming, such as motivation for playing [38, 39], and a relationship between PIU and comorbid psychiatric symptoms, such as depression [40]. There are also differences in the characteristics of gaming, such as playing frequency and game genre, between the adolescent and adult Internet game players [41]. A total of 60 unrelated participants (30 male out-patients with IGD [aged 22–42 years] and 30 sex-matched normal controls (NC) [aged 20–38 years]) were enrolled for a case-control study of IGD. IGD group participants with past or current medical disorders, neurological disorders, or other psychiatric disorders were excluded. NC with no history of medical, neurological, or psychiatric disorders were recruited from the general population. This study was conducted after written consent was obtained from all participants and was approved by the Institutional Review Board (IRB) of Seoul St. Mary’s Hospital (IRB number: KC12ONMI0377).

#### Measures

The participants answered questions in regard to age, years of education, marital status, and employment status. In addition, all participants were assessed for their history of alcohol and cigarette use, the Alcohol Use Disorders Identification Test (AUDIT), Fagerstrom test for Nicotine Dependence (FTND), Beck Depression Inventory (BDI), Beck Anxiety Inventory (BAI), and Barratt’s Impulsiveness Scale-11 (BIS-11). The characteristics of IGD were measured by the main purpose of Internet use, weekday/weekend average Internet gaming usage hours, and the Korean version of Young’s Internet Addiction Test (Y-IAT).

The Y-IAT is used worldwide to screen for PIU [42]. The Y-IAT was developed based on the DSM-IV diagnostic criteria for pathological gambling and is composed of 20 items with 5 Likert scales ranging from 1 (Not at all) to 5 (Always). The total scores range from 20 to 100, with the higher scores indicating a greater tendency towards addiction. The Y-IAT scores of 20–39 are regarded as an average user, 40–69 as a possible addicted user, and more than 70 as an addicted user [42].

In this study’s sample, Cronbach’s alphas were .89, .89, .93, .93, .99, and .98 for the AUDIT, FTND, BDI, BAI, BIS-11, and Y-IAT, respectively.

#### Target selection

As mentioned previously, the mesolimbic DA pathway is thought to play a key role in the development of addictions [28, 29], and serotonergic, glutamate, GABA, noradrenergic, cholinergic, and opioid neurotransmitter systems have been shown to interact with the mesolimbic DA pathway and modulate its activity [30, 31]. To date, genetic studies of IGD have shown an association between IGD and a genetic variation in a particular neurotransmitter-related gene. Therefore, we selected seventy-two individual genes, including genes of seven neurotransmitter (DA, serotonin [5-HT], glutamate, GABA, norepinephrine [NE], acetylcholine [Ach], and opioid) receptors, transporters, or synthesis and degradation enzymes. The full list of target genes is provided in the Supporting Information (S1 Table).

#### Target gene captured library construction, sequencing, and sequence analysis

We generated exome libraries that were enriched for the target genes’ coding sequences using the SureSelect capture probes from Agilent Technologies (Santa Clara, CA, USA). Each library was prepared according to the manufacturer’s recommendations (Illumina, San Diego, CA, USA). Genomic deoxyribonucleic acid (DNA) was extracted from the peripheral blood and fragmented. Following end-repair, Illumina adapters were ligated to the fragments. The sample was selected by aiming for a 350 to 400 base pair product and the selected product was amplified via polymerase chain reaction (PCR). The final product was validated using an Agilent Bioanalyzer (Agilent Technologies, Santa Clara, CA, USA).

Next, the validated and enriched libraries were loaded into a HiSeq2000 (Macrogen Facility, Seoul, Korea) for sequencing. The sequences produced by HiSeq2000 were mapped to the human genome, using the University of California Santa Cruz human genome (UCSC hg19) as the reference sequence and the mapping program Burrows-Wheeler Aligner (BWA, version 0.5.9rc1) [43]. Subsequently, we applied the programs packaged in Picard-tools (version 1.59; http://www.broadinstitute.org) to remove PCR duplicates and eliminate the reads not across the targeted exonic regions. The variants (single nucleotide polymorphisms [SNPs] and insertions and deletions [indels]) were called on each sample with the GATK Haplotype Caller (version 3.4.46; http://www.broadinstitute.org). The called variants were functionally annotated by ANNOVAR (version 2016; http://www.openbioinformatics.org).

#### Statistical analysis

Differences in demographic and clinical variables between the IGD and NC groups were tested using the Mann-Whitney U test or Chi-square test. The allelic association was tested using PLINK software (version 1.07) [44]. Some variants were filtered out using the Hardy-Weinberg equilibrium test (*p* < 1.0 X 10^−5^) and minor allele frequency (MAF) thresholds (MAF < 0.001). To analyze the associations, the allelic distributions between IGD and NC were compared using Fisher’s exact test. The *p*-values were adjusted using the genomic-control correction method [45]. Mean Y-IAT scores and weekday/weekend average Internet gaming usage hours of the groups with and without the allele that were significant between IGD and NC were compared using the Mann-Whitney U test. Statistical analyses were performed using SPSS version 24.0 (IBM Corp., Armonk, NY, USA), and *p*-values less than .05 were considered statistically significant.

### Study 2 (Case-control study of AD)

#### Participants

The AD patients were recruited from Incheon Chamsarang Hospital and were diagnosed according to the DSM-IV, Text Revision (DSM-IV-TR) criteria for AD by a clinically experienced psychiatrist (Y.H.C). For homogeneity, the participants were restricted to adult males. A total of 60 unrelated participants (31 male in-patients with AD [age range 37 to 57 years] and 29 male NC [age range 40 to 49 years]) were enrolled in a case-control study of AD. The participants of the AD group with past or current major medical disorders, neurological disorders, or other psychiatric disorders were excluded. NC with no history of medical, neurological, or psychiatric disorders were recruited from the local community. This study was approved by the IRB of Seoul St. Mary’s Hospital (IRB number: KC13ONMI0171). All participants provided written informed consent before participation.

#### Measures

Participants completed questions in regards to age, years of education, marital status, and employment status. In addition, all participants were assessed for their history of alcohol and cigarette use. BDI, BAI, and BIS-11 were applied and severity of AD was measured using AUDIT. In this study’s sample, Cronbach’s alphas were .95, .98, .97, and .94 for the BDI, BAI, BIS-11, and AUDIT, respectively.

#### Target selection

The same 72 genes that were included in study 1 and ten additional genes related to alcohol-metabolic enzyme were selected. The ten additional genes were alcohol dehydrogenase (*ADH*) *1A*, *ADH1B*, *ADH1C*, *ADH4*, *ADH5*, *ADH6*, *ADH7*, aldehyde dehydrogenase (*ALDH*) *1A1*, *ALDH2*, and cytochrome P450 (*CYP*) *2E1*.

#### Target gene captured library construction, sequencing, and sequence and statistical analysis

The methods of captured library construction, sequencing, bioinformatics, and statistical analysis were identical to those of study 1.

## Results

### Study 1 (Case-control study of IGD)

The demographic and clinical characteristics of the IGD patients and control participants are presented in Table 1. In the IGD group, the mean age was 30.73 ± 6.72 years, and the average playing duration on a weekday and weekend was 2.85 ± 1.74 hours and 4.50 ± 2.51 hours, respectively. The Y-IAT score of IGD patients was 55.60 ± 9.28, which is considered a possible addicted user. There were no differences in mean age, years of education, marital status, employment condition, history of smoking and alcohol use, and AUDIT and FTND scores between the two groups. However, the average Internet game usage hours during the weekday and weekend were significantly longer and the mean Y-IAT, BDI, BAI, and BIS-11 scores were significantly higher in the IGD group than in the NC group.

**Table 1:** Demographics and clinical characteristics of Korean male participants in study 1.

Variables	IGD (N = 30)	NC (N = 30)	χ2 or Mann-Whitney U	Z scores	*p*-value	Cohen’s *d* or Cramer’s V
Age, Mean years (SD)	30.73 (6.72)	30.57 (5.48)	448.00	-0.030	.976	0.026
Years of education, Mean years (SD)	15.47 (1.81)	14.97 (3.73)	443.00	-0.113	.910	0.171
Marital status, N (%)			1.15		.284	0.138
Unmarried	21 (70.0)	17 (56.7)				
Married	9 (30.0)	13 (43.3)				
Employed, N (%)			0.3		.584	0.071
Yes	19 (63.3)	21 (70.0)				
No	11 (36.7)	9 (30.0)				
Smoking, N (%)			0.34		.559	0.075
Non-smoker	21 (70.0)	23 (76.7)				
Current smoker	9 (30.0)	7 (23.3)				
Alcohol, N (%)			0.09		.766	0.038
Non-drinker	7 (23.3)	8 (26.7)				
Drinker	23 (76.7)	22 (73.3)				
AUDIT, Mean (SD)	15.00 (6.92)	14.20 (5.31)	424.00	-0.389	.697	0.130
FTND, Mean (SD)	1.50 (2.36)	1.27 (2.36)	430.50	-0.371	.710	0.097
Weekday average Internet game usage hours, Mean hours/day (SD)	2.85 (1.74)	1.65 (1.33)	226.00	-3.405	.001*	0.775
Weekend average Internet game usage hours, Mean hours/days (SD)	4.50 (2.51)	2.60 (1.99)	243.00	-3.094	.002*	0.839
Y-IAT, Mean (SD)	55.60 (9.28)	30.00 (5.78)	21.50	-6.348	< .001*	3.311
BDI, Mean (SD)	24.47 (10.73)	13.76 (9.58)	209.50	-3.558	< .001*	1.053
BAI, Mean (SD)	22.20 (11.33)	10.67 (9.31)	193.00	-3.807	< .001*	1.112
BIS-11, Mean (SD)	78.70 (14.00)	55.23 (22.01)	161.50	-4.285	< .001*	1.272

IGD, Internet gaming disorder patients; NC, Normal controls; SD, Standard deviation; AUDIT, Alcohol use disorders identification test; FTND, Fagerstrom test for nicotine dependence; Y-IAT, Young’s Internet addiction test; BDI, Beck depression inventory; BAI, Beck anxiety inventory; BIS-11, Barratt impulsiveness scale-11.

*p<0.05 in Mann-Whitney U test or Chi-square test

A total of 618 SNPs were identified in the IGD or NC group. These SNPs were then used for the association analysis. The average mean depth of the targeted regions among all samples was 757.69 ± 12.21-fold, and an average of 98.7% and 98.6% of the targeted bases were covered at 1- and 10-fold, respectively.

When the MAFs of the SNPs were compared between the IGD and NC groups, 16 variants had unadjusted *p*-values < .05. However, only one SNP, rs1044396 of nicotinic acetylcholine receptor alpha 4 subunit (*CHRNA4*) [corrected *p* = .043, odds ratio (OR) = 0.21], remained significant after correction of the *p* level and this is a synonymous SNP. The IGD group had a significantly lower number of T alleles of *CHRNA4* rs1044396 when compared to that of the controls; this variant had an OR < 1, which suggests a protective effect against IGD. Table 2 shows the characteristics and results of the allelic association analysis for the significant SNP rs1044396 of *CHRNA4*. The genotypic association test could not be performed because only one participant in the IGD group had a TT genotype.

**Table 2:** Characteristics and results of the allelic association analysis of the SNP showing a statistically significant difference in MAF between the IGD and NC groups.

SNP	Gene	Chromosome No.	Region	Major allele	Minor allele	MAF (IGD)	MAF (NC)	χ2	*p*	*p*′	Cramer’s V	OR (95% CI)
rs1044396*	*CHRNA4*	20	exon	C	T	0.0833	0.3	9.09	0.003	0.043	0.275	0.21 (0.07–0.62)

SNP, Single nucleotide polymorphism; MAF, Minor allele frequency; IGD, Internet gaming disorder patients; NC, Normal controls; No.,Number;

*p*, unadjusted *p*-value from Fisher’s exact test; *p*′, Genomic-control corrected *p*-value; OR, Odds ratio; CI, Confidence interval;

*CHRNA4*, Cholinergic receptor nicotinic alpha 4 subunit;

* synonymous SNP

When the entire sample was grouped into T–(CC genotype) and T+ groups (CT and TT genotype) according to presence or absence of the T allele of *CHRNA4* rs1044396, the Y-IAT score was significantly lower in the T+ group. This difference in Y-IAT remained significant after controlling for the BDI, BAI, and BIS-11 scores (Table 3).

**Table 3:** Comparison of clinical variables according to the genotypes of rs1044396 of *CHRNA4*.

Genotypes of *CHRNA4*	T- group (CC genotype)		T+ group (CT and TT genotype)		*p*	*p*′
	N = 38		N = 22			
	M	SD	M	SD		
BDI	19.50	11.61	18.44	11.37	.707	
BAI	16.84	11.63	15.73	12.37	.753	
BIS-11	68.63	22.49	64.09	20.66	.313	
Y-IAT	46.74	15.07	36.00	12.53	.008*	.003**
Weekday average Internet gaming-usage, Hours	2.43	1.85	1.93	1.20	.246	.348
Weekend average Internet gaming-usage, Hours	3.66	2.78	3.36	1.75	.914	.860

*p<0.05 in Mann-Whitney U test

**p′<0.05 when controlling for BDI, BAI, and BIS-11 scores

*CHRNA4*, Cholinergic receptor nicotinic alpha 4 subunit; BDI, Beck depression inventory; BAI, Beck anxiety inventory; BIS-11, Barratt impulsiveness scale-11; Y-IAT, Young’s Internet addiction test; M, Mean; SD, Standard deviation

### Study 2 (Case-control study of AD)

The demographic and clinical features of the AD patients and control participants are shown in Table 4. There was no difference in mean age and marital status between the two groups. However, the proportion of unemployment and current smokers, frequency and amount of alcohol drinking, and AUDIT, BDI, BAI, and BIS-11 scores were significantly higher and years of education were significantly shorter in the AD group when compared to those of the NC group.

**Table 4:** Demographics and clinical characteristics of Korean male participants in study 2.

Variables	AD	NC	χ2 or Mann-Whitney U	Z scores	*p*-value	Cohen’s *d* or Cramer’s V
	(N = 31)	(N = 29)				
Age, Mean years (SD)	45.23 (4.39)	44.79 (2.24)	438.00	-0.172	.864	0.126
Years of education, Mean years (SD)	13.81 (2.12)	15.45 (1.96)	249.50	-3.001	.003*	0.803
Marital status, N (%)			0.04		.833	0.027
Unmarried	12 (38.7)	12 (41.4)				
Married	19 (61.3)	17 (68.6)				
Employed, N (%)			19.29		< .001*	0.567
Yes	7 (22.6)	23 (79.3)				
No	24 (77.4)	6 (20.7)				
Smoking, N (%)			16.85		< .001*	0.530
Non-smoker	0 (0.0)	11 (37.9)				
Current smoker	29 (93.5)	14 (48.3)				
Ex-smoker	2 (6.5)	4 (13.8)				
Alcohol history						
Age at first alcohol use, Mean years (SD)	19.06 (1.36)	18.76 (0.83)	334.00	-1.783	.075	0.266
Frequency, days/week (SD)	4.53 (1.29)	0.67 (0.33)	0.00	-6.717	< .001*	4.100
Amount, Soju^a^ bottles/day (SD)	2.71 (1.19)	0.75 (0.29)	35.50	-6.207	< .001*	2.263
Presence of AUD family history, N (%)	9 (29.0)	2 (6.9)	4.90		.027*	0.286
AUDIT, Mean (SD)	27.20 (7.30)	5.70 (2.21)	1.00	-6.648	< .001*	3.986
BDI, Mean (SD)	19.23 (12.91)	6.72 (3.92)	197.50	-3.740	< .001*	1.311
BAI, Mean (SD)	16.42 (16.39)	6.62 (7.02)	289.00	-2.386	.017*	0.777
BIS-11, Mean (SD)	71.00 (2.94)	46.83 (5.87)	0.00	-6.720	< .001*	5.207

AD, Alcohol dependent patients; NC, Normal controls; SD, Standard deviation; AUDIT, Alcohol use disorders identification test; BDI, Beck depression inventory; BAI, Beck anxiety inventory; BIS-11, Barratt impulsiveness scale-11

*p<0.05 in Mann-Whitney U test or Chi-square test

^a:^ alcoholic beverage with alcohol concentration in the range of 19–22%

A total of 689 SNPs were found in the AD or NC groups. These SNPs were then used for the association analysis. The average mean depth for the targeted regions among all samples was 694.67 ± 83.51-fold. An average of 98.7% and 98.7% of targeted bases were covered at 1- and 10-fold, respectively.

When the MAFs of the SNPs were compared between the AD and NC groups, 28 variants had an unadjusted *p*-value < .05. In addition, two SNPs, rs1229984 [corrected *p* = .008, OR = 0.21] of *ADH1B* and rs671 [corrected *p* = .020, OR = 0.06] of *ALDH2*, remained significant after correction of the *p* level; all were non-synonymous SNPs.

The individuals in the AD group had a significantly lower number of the A alleles of *ADH1B* rs1229984 (ADH1B*2) and the A alleles of *ALDH2* rs671 (ALHD2*2) when compared with that of the NC group. The characteristics and the results of the allelic association analysis for the significant SNPs are shown in Table 5.

**Table 5:** Characteristics and results of the allelic association analysis of SNPs showing statistically significant difference in MAF between the AD and NC groups.

SNP	Gene	Chromosome No.	Region	Major allele	Minor allele	MAF (AD)	MAF (NC)	χ2	*p*	*p*′	Cramer’s V	OR (95% CI)
rs1229984†	*ADH1B*	4	exon	G	A	0.4516	0.7931	14.78	<0.001	0.008	0.351	0.21 (0.10–0.48)
rs671†	*ALDH2*	12	exon	G	A	0.0161	0.2069	11.29	0.001	0.020	0.307	0.06 (0.01–0.50)

SNP, Single nucleotide polymorphism; MAF, Minor allele frequency; AD, Alcohol dependent patients; NC, Normal controls; *p*, unadjusted *p*-value from Fisher’s exact test; *p*′, Genomic-control corrected *p*-value; OR, Odds ratio; CI, Confidence interval;*ADH1B*, Alcohol dehydrogenase 1B; *ALDH2*, Aldehyde dehydrogenase 2;

^†^non-synonymous SNP.

## Discussion

Our primary aim of the present study was to investigate the genetic predisposition of IGD with the secondary aim of comparing the results to those with AD. We performed a case-control association study for IGD in Korean male adults using 618 SNPs located in 72 candidate genes that encode addiction-associated neurotransmitter systems. In addition, we conducted a case-control study for AD using the same candidate genes and the same method (TES). Consequently, we found that the marker rs1044396 (*CHRNA4*) was significantly associated with IGD. The IGD group had lower numbers of the T allele of rs1044396, and this variant represents a trend of protective effect (OR, 0.21; 95% CI, 0.07–0.62) against IGD. Moreover, the T+ group (CT and TT genotype) of rs1044396 had lower Y-IAT scores when compared to that of the T– group (CC genotype). This finding is consistent with the result of a German case-control study of 132 Internet addicts (Y-IAT scores higher than 39) and 132 controls [17]. The German study reported that the CC genotype (T– variant) of the rs1044396 polymorphism on the *CHRNA4* gene occurred more frequently in the case group [17]. In other words, our study replicated the findings of the *CHRNA4* polymorphism association with IGD in a sample of German adults.

Although there have been few studies on the neurobiology of IGD, dopaminergic systems influencing reward and reinforcing behaviors have been implicated in IGD [14, 46, 47]. A study using single photon emission computed tomography (SPECT) suggested that the level of dopamine release in the ventral striatum during a motorcycle riding computer game is comparable to that induced by addictive substances [14, 48]. Additionally, a previous case-control study of seventy-nine male adolescents with excessive Internet video game play (played Internet video games more than 1 hour/day and a Y-IAT score higher than 50) and 75 healthy adolescents showed that the Taq1A1 allele of dopamine receptor gene (*DRD2*) was associated with excessive game play; individuals with this allele scored higher on a reward-dependence scale [15].

Another association study that examined the genetic polymorphisms of the serotonin transporter gene (*5-HTTLPR*) showed that 91 male adolescents with excessive Internet use (used the Internet more than 1 hour/day and a Y-IAT score higher than 50) had a higher homozygous short allelic variant of 5-*HTTLPR* frequencies and BDI scores when compared to that of 75 healthy participants [16].

We also investigated dopaminergic and serotonergic system-related genes, including *DRD2* and *5-HTTLPR* as target genes, but we did not find allelic differences within the dopaminergic and serotonergic pathway genes between the case and control groups. This may be partially due to differences in participants and the selection criteria for the case group. We recruited young male adults rather than adolescents and the participants in the case group were examined by direct interview according to the DSM-5 criteria for IGD instead of using questionnaire scores.

The present study identified the influence of the gene coding for the alpha4 subunit of the nicotinic acetylcholine receptor (*CHRNA4*) on IGD. With respect to addiction, a biological link between *CHRNA4* and the nicotine-addiction phenotype has been established [21, 49–51]. Knock-in mice with a point mutation in the *CHRNA4* gene showed reduced nigrostriatal dopaminergic function [52]. Family-based association testing showed that rs1044396 of the *CHRNA4* gene was significantly associated with a protective effect against nicotine addiction [21], similar to our results. One possible explanation for the effect of the *CHRNA4* gene on addiction is that the cholinergic pathway is involved in actions in the mesolimbic dopaminergic system, which serves a principal role in the acquisition of addictive behavior [53]. Nearly all ventral tegmental area (VTA) DA neurons display an Ach-induced current with α4β2-type nicotinic acetylcholine receptors (nAchRs) and the activation of the α4 subunit is especially important for the modulation of nicotine-induced reward, sensitization, and tolerance [54].

Furthermore, previous studies have shown a significant association between polymorphisms in *CHRNA4* and the psychological risk attitude, response inhibition, and harm avoidance [55, 56]. Impaired response inhibition is an important issue in behavioral addiction [57–59] and previous works have revealed significant associations between harm avoidance and pathological gambling [60–62].

These combined results suggest that the nicotinic acetylcholine receptor may play a role in IGD by modulating the dopaminergic pathway and may affect the addiction phenotype, such as response inhibition. There were no differences in BDI, BAI, and BIS-11 scores, regardless of whether participants have the T allele of rs1044396 *CHRNA4*, but Y-IAT remained significant after controlling for these scores.

We also conducted an independent case-control association study for AD using the same 72 candidate genes included in study 1 and ten additional alcohol-metabolizing enzyme genes. The participants of study 2 were in-patients and years of education were shorter than those of the control group. In addition, the proportions of unemployment and current smokers were higher than those of the control group. According to previous studies, the prevalence of AD is negatively associated with years of education [63] and the unemployment rates in alcohol treatment programs are remarkably high [64]. Additionally, smoking is more common in the treatment-seeking alcoholic participants than in the non-alcoholic participants [65] and there appeared to be overlap in the genetic factors that influence both conditions, such as the genes that encode receptors for the neurotransmitter GABA [66]. However, we did not find any significant differences in the polymorphisms of the 72 genes that encode neurotransmitter systems between the two groups. Two polymorphisms in the alcohol-metabolizing enzyme gene were identified. The frequencies of the ADH1B*2 (rs1229984) and ALDH2*2 (rs671) alleles were significantly lower in AD than in NC, which is consistent with the results of previous studies [34, 67]. Multiple studies have reported lower frequencies of both the ADH1B*2 and ALDH2*2 alleles in alcoholic participants when compared to that of nonalcoholics participants in a variety of East Asian populations [68–72].

Few studies have compared IGD and substance addictions and comparisons of genetic studies are limited. The existing data suggest that IGD frequently co-occurs with substance addiction [73, 74], and this co-morbidity suggests a common underlying genetic vulnerability for IGD and substance addiction, including AD. To address this assumption, the present study conducted two case-control studies of IGD and AD using the same candidate genes, but the results do not support the assumption. Although rs1044396 of the *CHRNA4* gene was associated with nicotine addiction and the addiction phenotype [21, 49–51, 55], further association, family-aggregation, or twin studies are needed to examine the extent to which IGD shares a common genetic vulnerability with substance addictions.

This study has the following limitations. First, our sample size was small, and the study was limited to males. In addition, the genotypic association test could not be performed because only one participant in the IGD group had the TT genotype. Although we used a new method to identify the genetic variants and males are more likely to be addicted to the Internet games than females [75], the results may not be completely generalizable. Second, we did not have a replication sample. While our results are consistent with those of a German case-control association study, a replication of this study using a larger, independent sample with females is essential to validate the results. Third, we compared the results of two independent case-control studies that did not use the same control group because of age differences between IGD and AD patients, although we utilized the same method and the same candidate genes. Fourth, there are some limitations to the measurement of the Y-IAT. Though the Y-IAT is one of the most common instruments used to assess problematic Internet use and had good internal consistency in this study, the composition of questions are not limited to Internet gaming and some items have conflicting results in regards to its psychometric properties [76].

Despite these limitations, this study demonstrated that rs1044396 in exon 5 of *CHRNA4* is significantly associated with a protective effect against IGD and a quantitative phenotype (Y-IAT), which was consistent with the results of a German case-control study. To the best of our knowledge, the present investigation is the first study to replicate positive findings in a genetic association study of IGD diagnosed via the DSM-5 criteria and to compare the results of substance addiction and IGD under the several neurotransmitter system genes that have been implicated in the pathogenesis of addiction.

In conclusion, this study showed that a SNP (rs1044396 of *CHRNA4*) was significantly associated with IGD based on a case-control study using a sample of Korean male adults. The IGD was associated with a lower frequency of the T allele of rs1044396, and the CT and TT genotypes referred to lower addiction severity. These results suggest that the cholinergic system may be involved in the pathogenesis of IGD similar to substance addictions. Subsequent studies are needed to clarify the role of this promising gene in the pathogenesis and genetics of IGD.

## Supporting information

S1 Table Seventy-two target genes used in both study 1 and study 2. (DOCX)

S1 File Raw data. (XLSX)
