# Supplementary material for: The association between the nicotinic acetylcholine receptor α4 subunit gene (CHRNA4) rs1044396 and Internet gaming disorder in Korean male adults
Source: PLoS One. 2017 Dec 14;12(12):e0188358. doi: 10.1371/journal.pone.0188358 (PMC5730169; doi:10.1371/journal.pone.0188358)
Supplement: S1 Table — (DOCX) [file pone.0188358.s001.docx]

**S1 Table. Seventy-two target genes used in both Study 1 and Study 2**

| Neurotransmitters | Gene symbol | Gene full name | Ensemble | Chromosome |
| --- | --- | --- | --- | --- |
| Dopamine | DRD1 | Dopamine receptor D1 | [ENSG00000184845](http://www.ensembl.org/Homo_sapiens/geneview?gene=ENSG00000184845) | 5 |
|  | DRD2 | Dopamine receptor D2 | [ENSG00000149295](http://www.ensembl.org/id/ENSG00000149295) | 11 |
|  | DRD3 | Dopamine receptor D3 | [ENSG00000151577](http://www.ensembl.org/id/ENSG00000151577) | 3 |
|  | DRD4 | Dopamine receptor D4 | [ENSG00000069696](http://www.ensembl.org/id/ENSG00000069696) | 11 |
|  | DRD5 | Dopamine receptor D5 | [ENSG00000169676](http://www.ensembl.org/id/ENSG00000169676) | 4 |
|  | ANKK1 | Ankyrin repeat and kinase domain containing 1 | [ENSG00000170209](http://www.ensembl.org/id/ENSG00000170209) | 11 |
|  | SLC6A3 | Solute carrier family 6 number 3 | [ENSG00000142319](http://www.ensembl.org/id/ENSG00000142319) | 5 |
|  | SLC18A1 | Solute carrier family 18 number A1 | [ENSG00000036565](http://www.ensembl.org/id/ENSG00000036565) | 8 |
|  | SLC18A2 | Solute carrier family 18 number A2 | [ENSG00000165646](http://www.ensembl.org/id/ENSG00000165646) | 10 |
|  | TH | Tyrosine hydroxylase | [ENSG00000180176](http://www.ensembl.org/id/ENSG00000180176) | 11 |
|  | DDC | Dopa decarboxylase | [ENSG00000132437](http://www.ensembl.org/id/ENSG00000132437) | 7 |
|  | COMT | Catechol-O-methyltransferase | [ENSG00000093010](http://www.ensembl.org/id/ENSG00000093010) | 22 |
|  | MAOB | Monoamine oxidase B | [ENSG00000069535](http://www.ensembl.org/id/ENSG00000069535) | X |
| Glutamate | GRIN1 | Glutamate ionotropic receptor NMDA type subunit 1 | [ENSG00000176884](http://www.ensembl.org/id/ENSG00000176884) | 9 |
|  | GRIN2A | Glutamate ionotropic receptor NMDA type subunit 2A | [ENSG00000183454](http://www.ensembl.org/id/ENSG00000183454) | 16 |
|  | GRIN2B | Glutamate ionotropic receptor NMDA type subunit 2B | [ENSG00000273079](http://www.ensembl.org/id/ENSG00000273079) | 12 |
|  | GRIN2C | Glutamate ionotropic receptor NMDA type subunit 2C | [ENSG00000161509](http://www.ensembl.org/id/ENSG00000161509) | 17 |
|  | GRIK1 | Glutamate ionotropic receptor NMDA type subunit 1 | [ENSG00000171189](http://www.ensembl.org/id/ENSG00000171189) | 21 |
|  | GRM2 | Glutamate metabotropic receptor 2 | [ENSG00000164082](http://www.ensembl.org/id/ENSG00000164082) | 3 |
|  | GLRA1 | Glycine receptor alpha 1 | [ENSG00000145888](http://www.ensembl.org/id/ENSG00000145888) | 5 |
|  | GLRA2 | Glycine receptor alpha 2 | [ENSG00000101958](http://www.ensembl.org/id/ENSG00000101958) | X |
|  | GLRB | Glycine receptor beta | [ENSG00000109738](http://www.ensembl.org/id/ENSG00000109738) | 4 |
| GABA | GABRA2 | GABA type A receptor alpha2 subunit | [ENSG00000151834](http://www.ensembl.org/id/ENSG00000151834) | 4 |
|  | GABRA3 | GABA type A receptor alpha3 subunit | [ENSG00000011677](http://www.ensembl.org/id/ENSG00000011677) | X |
|  | GABRA4 | GABA type A receptor alpha4 subunit | [ENSG00000109158](http://www.ensembl.org/id/ENSG00000109158) | 4 |
|  | GABRA5 | GABA type A receptor alpha5 subunit | [ENSG00000186297](http://www.ensembl.org/id/ENSG00000186297) | 15 |
|  | GABRA6 | GABA type A receptor alpha6 subunit | [ENSG00000145863](http://www.ensembl.org/id/ENSG00000145863) | 5 |
|  | GABRB1 | GABA type A receptor beta1 subunit | [ENSG00000163288](http://www.ensembl.org/id/ENSG00000163288) | 4 |
|  | GABRB2 | GABA type A receptor beta2 subunit | [ENSG00000145864](http://www.ensembl.org/id/ENSG00000145864) | 5 |
|  | GABRB3 | GABA type A receptor beta3 subunit | [ENSG00000166206](http://www.ensembl.org/id/ENSG00000166206) | 15 |
|  | GABRD | GABA type A receptor delta subunit | [ENSG00000187730](http://www.ensembl.org/id/ENSG00000187730) | 1 |

GABA: Gamma-aminobutyric acid, ENSG: Ensemble (genome browser) identifiers

**S1 Table (continued). Seventy-two target genes used in both Study 1 and Study 2**

| Neurotransmitters | Gene symbol | Gene full name | Ensemble | Chromosome |
| --- | --- | --- | --- | --- |
| GABA | GABRG1 | GABA type A receptor gamma1 subunit | [ENSG00000163285](http://www.ensembl.org/Homo_sapiens/geneview?gene=ENSG00000163285;db=core) | 4 |
|  | GABRG2 | GABA type A receptor gamma2 subunit | [ENSG00000113327](http://www.ensembl.org/Homo_sapiens/geneview?gene=ENSG00000113327;db=core) | 5 |
|  | GABRG3 | GABA type A receptor gamma3 subunit | [ENSG00000182256](http://www.ensembl.org/Homo_sapiens/geneview?gene=ENSG00000182256;db=core) | 15 |
|  | GABBR1 | GABA type B receptor subunit1 | [ENSG00000204681](http://www.ensembl.org/id/ENSG00000204681) | 6 |
|  | SLC6A7 | Solute carrier family 6 number 7 | [ENSG00000011083](http://www.ensembl.org/id/ENSG00000011083) | 5 |
|  | SLC6A11 | Solute carrier family 6 number 11 | [ENSG00000132164](http://www.ensembl.org/id/ENSG00000132164) | 3 |
|  | SLC6A13 | Solute carrier family 6 number 13 | [ENSG00000010379](http://www.ensembl.org/id/ENSG00000010379) | 12 |
|  | SLC32A1 | Solute carrier family 32 number 1 | [ENSG00000101438](http://www.ensembl.org/id/ENSG00000101438) | 20 |
|  | GAD1 | Glutamate decarboxylase 1 | [ENSG00000128683](http://www.ensembl.org/id/ENSG00000128683) | 2 |
|  | GAD2 | Glutamate decarboxylase 2 | [ENSG00000136750](http://www.ensembl.org/id/ENSG00000136750) | 10 |
| Serotonin | HTR1A | 5-Hydroxytryptamine receptor 1A | [ENSG00000178394](http://www.ensembl.org/id/ENSG00000178394) | 5 |
|  | HTR1B | 5-Hydroxytryptamine receptor 1B | [ENSG00000135312](http://www.ensembl.org/id/ENSG00000135312) | 6 |
|  | HTR2A | 5-Hydroxytryptamine receptor 2A | [ENSG00000102468](http://www.ensembl.org/id/ENSG00000102468) | 13 |
|  | HTR2B | 5-Hydroxytryptamine receptor 2B | [ENSG00000135914](http://www.ensembl.org/id/ENSG00000135914) | 2 |
|  | HTR2C | 5-Hydroxytryptamine receptor 2B | [ENSG00000147246](http://www.ensembl.org/id/ENSG00000147246) | X |
|  | HTR3A | 5-Hydroxytryptamine receptor 3A | [ENSG00000166736](http://www.ensembl.org/id/ENSG00000166736) | 11 |
|  | HTR3B | 5-Hydroxytryptamine receptor 3B | [ENSG00000149305](http://www.ensembl.org/id/ENSG00000149305) | 11 |
|  | SLC6A4 | Solute carrier family 6 number 4 | [ENSG00000108576](http://www.ensembl.org/id/ENSG00000108576) | 17 |
|  | TPH1 | Tryptophan hydroxylase 1 | [ENSG00000129167](http://www.ensembl.org/id/ENSG00000129167) | 11 |
|  | TPH2 | Tryptophan hydroxylase 2 | [ENSG00000139287](http://www.ensembl.org/id/ENSG00000139287) | 12 |
|  | MAOA | Monoamine oxidase A | [ENSG00000189221](http://www.ensembl.org/id/ENSG00000189221) | X |
| Norepinephrine | ADRA1A | Adrenoceptor alpha 1A | [ENSG00000120907](http://www.ensembl.org/id/ENSG00000120907) | 8 |
|  | ADRA2A | Adrenoceptor alpha 2A | [ENSG00000150594](http://www.ensembl.org/id/ENSG00000150594) | 10 |
|  | ADRA2B | Adrenoceptor alpha 2B | [ENSG00000274286](http://www.ensembl.org/id/ENSG00000274286) | 2 |
|  | ADRA2C | Adrenoceptor alpha 2C | [ENSG00000184160](http://www.ensembl.org/id/ENSG00000184160) | 4 |
|  | ADRB2 | Adrenoceptor beta 2 | [ENSG00000169252](http://www.ensembl.org/id/ENSG00000169252) | 5 |
|  | SLC6A2 | Solute carrier family 6 number 2 | [ENSG00000103546](http://www.ensembl.org/id/ENSG00000103546) | 16 |
|  | DBH | Dopamine beta-hydroxylase | [ENSG00000123454](http://www.ensembl.org/id/ENSG00000123454) | 9 |
| Acetylcholine | CHRNA2 | Cholinergic receptor nicotinic alpha 2 subunit | [ENSG00000120903](http://www.ensembl.org/id/ENSG00000120903) | 8 |
|  | CHRNA3 | Cholinergic receptor nicotinic alpha 3 subunit | [ENSG00000080644](http://www.ensembl.org/id/ENSG00000080644) | 15 |
|  | CHRNA4 | Cholinergic receptor nicotinic alpha 4 subunit | [ENSG00000101204](http://www.ensembl.org/id/ENSG00000101204) | 20 |
|  | CHRNA5 | Cholinergic receptor nicotinic alpha 5 subunit | [ENSG00000169684](http://www.ensembl.org/id/ENSG00000169684) | 15 |
|  | CHRNB2 | Cholinergic receptor nicotinic beta 2 subunit | [ENSG00000160716](http://www.ensembl.org/id/ENSG00000160716) | 1 |
|  | CHRNB4 | Cholinergic receptor nicotinic beta 4 subunit | [ENSG00000117971](http://www.ensembl.org/id/ENSG00000117971) | 15 |
|  | CHRM2 | Cholinergic receptor muscarinic 2 | [ENSG00000181072](http://www.ensembl.org/id/ENSG00000181072) | 7 |

GABA: Gamma-aminobutyric acid, ENSG: Ensemble (genome browser) identifiers

**S1 Table (continued). Seventy-two target genes used in both Study 1 and Study 2**

| Neurotransmitters | Gene symbol | Gene full name | Ensemble | Chromosome |
| --- | --- | --- | --- | --- |
| Opioid | OPRM1 | Opioid receptor mu 1 | [ENSG00000112038](http://www.ensembl.org/id/ENSG00000112038) | 6 |
|  | OPRK1 | Opioid receptor kappa 1 | [ENSG00000082556](http://www.ensembl.org/id/ENSG00000082556) | 8 |
|  | OPRD1 | Opioid receptor delta 1 | [ENSG00000116329](http://www.ensembl.org/id/ENSG00000116329) | 1 |
|  | OPRL1 | Opioid related nociception receptor 1 | [ENSG00000125510](http://www.ensembl.org/id/ENSG00000125510) | 20 |
|  | PDYN | Prodynorphin | [ENSG00000101327](http://www.ensembl.org/id/ENSG00000101327) | 20 |
|  | PENK | Proenkephalin | [ENSG00000181195](http://www.ensembl.org/id/ENSG00000181195) | 8 |

ENSG: Ensemble (genome browser) identifiers
